# Support on four paws—does the integration of a therapy dog reduce anxiety and increase positive affect in spider phobics during in vivo exposure therapy?: study protocol for a parallel randomised controlled trial of two groups to compare one-session in vivo exposure treatment of spider phobia with and without a therapy dog

**DOI:** 10.1136/bmjopen-2025-101648

**Published:** 2025-07-15

**Authors:** Moritz Nicolai Braun, Tanja Michael, Monika Equit, Johanna Lass-Hennemann

**Affiliations:** 1 Department of Psychology, Saarland University, Saarbrücken, Germany

**Keywords:** Patients, Anxiety disorders, PSYCHIATRY

## Abstract

**Introduction:**

Exposure is a central component in the treatment of a range of mental disorders. However, despite high efficacy and efficiency, dissemination of exposure-based treatments is limited. Important factors that contribute to this limited dissemination are negative beliefs about exposure on the part of the public, the therapists, and the patients. While patients perceive exposure therapy as burdensome, therapists are concerned about putting too much strain on their patients during exposure, leading to suboptimal delivery of exposure. In a previous study, in which healthy participants underwent a differential fear conditioning paradigm, we found initial evidence that the integration of a therapy dog into exposure reduces participants’ anxiety and increases participants’ positive affect without causing poor treatment outcome. Thus, the integration of a therapy dog into exposure might be a promising approach to address patients’ and therapists’ concerns and, thus, to (1) foster dissemination of exposure that is (2) delivered in an optimal manner. To scrutinise our findings in a clinical sample, we designed the present study. We test the following hypotheses: (H1) participants in the dog group report significantly less anxiety during the course of the treatment than participants in the control group. (H2) Participants in the dog group report significantly more positive affect during the course of the treatment than participants in the control group. (H3) Participants in the dog group report significantly higher therapy motivation than participants in the control group. (H4) Participants in the dog group report significantly lower anticipatory anxiety than participants in the control group. (H5) The treatment in the dog group is not inferior to the treatment in the control group.

**Methods and analysis:**

In this parallel randomised controlled trial of two groups, *n*=88 participants (spider phobics without: a current diagnosis of a mental disorder other than a specific phobia, insect bite allergy, dog hair allergy, fear of dogs, current psychopharmacological treatment, and current psychotherapeutic treatment; the sample size calculation is based on the results from our previous study) are randomly allocated (with a 1:1 allocation as per a computer-generated randomisation schedule) to either an ambulant one-session in vivo exposure treatment of spider phobia with a therapy dog (dog group) or without a dog (control group). Due to the nature of the intervention, neither participants nor therapists can be blinded once participants are allocated to one of the two groups. However, the person conducting screening and diagnostics is blind to the allocation, participants are blind to the hypotheses and the respective other group, and the researchers are blind to the allocation while analysing the data. We will test (H1) and (H2), concerned with our primary outcomes, by means of 2×4 mixed analyses of variance with the between-subjects factor group (dog group vs. control group), the within-subjects factor time (with four levels, one for each time point anxiety and affect are measured during treatment), and anxiety or positive affect as the dependent variable, respectively. We will test (H3) and (H4) by means of an analyses of covariance with therapy motivation/anticipatory anxiety at baseline as the covariate, the between-subjects factor group (dog group vs. control group) and therapy motivation/anticipatory anxiety at pre-treatment as the dependent variable, respectively. We will test (H5) by means of 95% CIs and non-inferiority zones.

**Ethics and dissemination:**

This trial was approved by our university’s ethics committee (reference number 24–11). Any deviations from this study protocol or the preregistrations as well as any adverse events potentially arising in the course of the trial, will be made explicit in the publication of the trial results. All participants provided written informed consent prior to the inclusion into the trial. The findings from this trial will be disseminated by means of common academic pathways, including peer-reviewed publications and conference presentations. Following common open science practices, data and analysis code will also be made publicly available in anonymised form on the Open Science Framework (osf.io).

**Trial registration number:**

On 18 June 2024, this study was registered at the German Clinical Trials Register (ID: DRKS00034494; https://drks.de/search/de/trial/DRKS00034494) and preregistered at AsPredicted (https://aspredicted.org/JRP_SCF).

**STRENGTHS AND LIMITATIONS OF THIS STUDY**

- The present trial is rigorously designed, strictly follows state-of-the-art guidelines, and adheres to the principles of open and reproducible science.
- The randomised and controlled design of the present trial enhances internal validity by reducing biases and is the ‘gold standard’ of clinical research.
- The treatment for spider phobia we employ is well-established and manualised, which yields the possibility to investigate the effects of the integration of a dog while delivering the treatment in an optimal manner.
- Due to the nature of the intervention, neither participants nor therapists can be blinded once participants are allocated to one of the two groups.
- In its briefness, the one-session treatment employed in the present trial may not necessarily reflect typical clinical settings, where exposure therapy is commonly delivered over multiple sessions.

## Introduction

### Background and rationale

 Exposure—that is, confrontation of patients with those stimuli or situations that cause strong subjective discomfort (e.g., fear, disgust and tension) in an objectively safe environment^1^—is a central component in the treatment of a range of mental disorders, including anxiety disorders, trauma- and stressor-related disorders, obsessive-compulsive disorder and eating disorders.^2^,^7^ It has repeatedly and consistently been shown that exposure-based treatments are highly effective, with exposure-based treatments outperforming non-active, active, and placebo controls.^8^,^11^

However, despite their high efficacy and efficiency (effective treatment of specific phobias is possible in even a single session^12^,^14^), exposure-based treatments are by no means employed in all relevant cases. For instance, data from German samples suggest that exposure is only used in less than half of the behavioural therapeutic treatments of anxiety disorders^15^ andless than 30% of the behavioural therapeutic treatments of obsessive-compulsive disorders.^16^ This limited dissemination of exposure is similarly evident in data from North American and Dutch samples^17^,^19^ and thus, in different healthcare systems.

Which factors contribute to this limited dissemination of exposure? There is growing evidence that besides practical issues such as limited time for exposure therapy and unpredictable time management,^20^ the limited dissemination of exposure is associated with a range of negative beliefs on the part of the public, the therapists, and the patients. In terms of public opinion, researchers identified a ‘public relations problem’ for exposure therapy, as misinformation about exposure-based treatments has led to the misconception that exposure treatment is cruel, unethical, and overly burdensome for vulnerable people.^21 22^

Abundant research has shown that negative beliefs about exposure are also common among therapists. The most common ones are that exposure evokes too much distress for the patient, that exposure renders arousal reduction strategies necessary, and that exposure puts the patient at risk of decompensating.^20 23^ Thus, therapists are concerned to put too much strain on their patients during exposure. Therapists react to this concern by delivering exposure, if at all, in an overly cautious and suboptimal manner, including the creation of a less ambitious exposure hierarchy, a less anxiety-provoking exposure task, and attempts to minimise patients’ anxiety during exposure.^24^ This has been conceptualised as therapist safety behaviours and argued to put the treatment at risk of a poor outcome.^23 25^

Even though studies on patients’ beliefs about exposure therapy (*prior* to the start of exposure therapy) are rare, there are a few findings showing that patients perceive exposure therapy as burdensome (see Richard and Gloster, 2007^22^). Moreover, one study shows that in a sample of patients with chronic post-traumatic stress disorder, patients significantly preferred other therapy forms over exposure, despite explanations emphasising exposure therapies’ greater empirical support.^26^ In line with this, our clinical experience shows that patients are reluctant towards exposure therapy and that patient engagement with exposure therapy requires much more support, motivation, and information than patient engagement with other cognitive behavioural therapeutic techniques.

To summarise, exposure is highly effective and efficient and a central component in the treatment of a range of mental disorders. However, this effective therapeutic technique is by no means applied in all relevant cases (at least partly) due to concerns on the patients’ and the therapists’ side. Thus, addressing these concerns might be a promising approach to (1) foster dissemination of exposure that is (2) delivered in an optimal manner.

One way of addressing patients’ and therapists’ concerns might be the integration of a therapy dog. Dog-assisted interventions—that is, interventions in which the interaction between humans and trained (therapy) dogs are a central component^27^—have received increasing attention in recent decades, with a range of positive effects on psychological and psychosocial variables reported in the literature. Relevantly, there is repeated evidence that dog-assisted interventions reduce anxiety and stress (for reviews see Beetz *et al.*, 2012 and Ein *et al.*, 2018^28 29^). For instance, healthy participants who were accompanied by a dog while watching a traumatic film clip reported less anxiety and less negative affect after watching the clip than participants accompanied by a toy dog and participants without a companion.^30^ Similarly, healthy participants who interacted with a dog for 15 min after watching a traumatic film clip reported less anxiety, less negative affect, and more positive affect than participants who, after watching the traumatic film clip, relaxed for 15 min without interacting with a dog.^31^ More specifically, in a previous study from our group,^32^ healthy female participants underwent a differential fear conditioning paradigm (i.e., a laboratory analogue to the development and treatment of an anxiety disorder). Crucially, the participants completed the extinction phase (i.e., a laboratory analogue to exposure therapy) either alone (control group), in the presence of a friendly female person (social support group) or in the presence of a therapy dog (dog group). Participants in the dog group, but not participants in the social support group, reported significantly reduced anxiety and significantly increased positive affect during extinction compared with participants in the control group. Importantly, while the participants in the dog group showed impaired learning during extinction compared with the control group, this difference vanished during reinstatement (i.e., the laboratory analogue of a new encounter with the fear-provoking stimulus). We interpret these results as initial evidence that the integration of a dog reduces anxiety and increases positive affect without causing poor treatment outcome. This latter aspect is especially important, since the integration of a dog might be seen as a therapist safety behaviour and is thus susceptible to the presumption that it leads to poor treatment outcome.^23^ However, in case it does not negatively influence the outcome of the treatment, given its positive effects on patients’ anxiety and affect, the integration of a dog appears well suited to address patients’ and therapists’ concerns and might thus be well suited to foster dissemination of exposure in an optimal manner.

To scrutinise our previous findings as well as our interpretation in a clinical sample, we designed the present study. In this parallel randomised controlled trial of two groups, participants are randomly allocated to either an ambulant one-session in vivo exposure treatment of spider phobia^12^,^14^ with a therapy dog or an ambulant one-session in vivo exposure treatment for spider phobia without a dog (the standard care). We opted for the clinical population of patients with spider phobia for three reasons. First, specific phobias of the animal type, especially spider phobia, have a high prevalence,^33^,^35^ making patients with spider phobia a population that is relatively easy to recruit. Second, with the availability of the one-session treatment, specific phobias in general and spider phobia in particular can be treated with high efficiency and efficacy in just a single, 3-hour session. This makes the treatment of spider phobia well suited and feasible in the course of a clinical trial. Third, the one-session treatment for spider phobia is manualised.^36^ Thus, the exposure hierarchy and the exposure task are set, which yields the possibility to investigate the effects of the integration of a dog while delivering the treatment in an optimal manner.

### Objectives

Given our previous findings and evidence that dog-assisted interventions increase motivation,^37^ we test the following hypotheses:

- (H1) Participants in the dog group report significantly less anxiety during the course of the treatment than participants in the control group.
- (H2) Participants in the dog group report significantly more positive affect during the course of the treatment than participants in the control group.
- (H3) Participants in the dog group report significantly higher therapy motivation than participants in the control group.
- (H4) Participants in the dog group report significantly lower anticipatory anxiety than participants in the control group.
- (H5) The treatment in the dog group is not inferior to the treatment in the control group.

In addition, we explore whether participants in the dog group report higher treatment satisfaction than participants in the control group and whether participants in the dog group report a better therapeutic alliance than participants in the control group. The latter seems especially interesting in light of the importance of the therapeutic alliance for the success of psychotherapy^38^ and ongoing research about whether and how the presence of a dog improves the therapeutic alliance.^39^,^41^ Importantly, differences in the attitude towards pets in general and dogs in particular, potentially due to differences in cultural or religious backgrounds, might influence the acceptance and effects of the integration of a dog into therapy. To be able to address this, we also collect data on the participants’ attitude towards pets.

## Methods and analysis

### Patient and public involvement

None.

### Trial design and setting

This study is a parallel randomised controlled trial of two groups (allocation ratio 1:1; unit of randomisation: individual participant) to compare one-session in vivo exposure treatment of spider phobia^12^,^14^ with a therapy dog (a trained, 7-year-old, light-coloured, medium-sized crossbreed; dog group) with one-session in vivo exposure treatment for spider phobia without a dog (control group). The study is conducted at the psychotherapy outpatient unit of our university. Participants are recruited from the general public.

### Eligibility criteria

#### Inclusion criteria

Eligible for inclusion are adults with spider phobia with a score of at least 14 in the Fear of Spider Screening (see the *Outcomes* section below) and a score of at least 50 in the Fear of Spiders Questionnaire (FSQ) (see the *Outcomes* section below) who do not fulfil any exclusion criteria.

#### Exclusion criteria

Exclusion criteria comprise the current diagnosis of a mental disorder other than a specific phobia (either already known to the participant prior to their participation in our study or diagnosed by means of the short version of the Diagnostic Interview for Mental Disorders (Mini-DIPS; see the *Additional measures* section below for details) during the course of the diagnostic interview), insect bite allergy, dog hair allergy, fear of dogs, current psychopharmacological treatment, and current psychotherapeutic treatment. All participants give their written informed consent to participate in the trial (see the online supplemental file 1).

### Intervention and comparator

#### Control group

Participants in the control group receive the regular one-session treatment for spider phobia developed by Öst in the 1980s,^12^,^14^ which consists of a 1-hour psychoeducation session and a 3-hour treatment session in which participants are gradually exposed to spiders of increasing sizes.

#### Dog group

Patients in the dog group receive the same one-session treatment for spider phobia as participants in the control group. However, a certified therapy dog is integrated into the first contact with the therapist, the psychoeducation, and the 3-hour treatment session. Details about the complete procedure, how exactly the dog is integrated, and how the two conditions differ are provided in the *Participant timeline* section below.

The interventions are provided by therapists who were trained by JLH, who herself has been trained by Lars-Göran Öst in preparation for a previous spider phobia study^42^ and who has frequently engaged in therapies for spider phobia ever since. In addition, each of the therapists completed at least one pilot treatment in the presence of JLH as well as one video-recorded pilot treatment that they subsequently discussed with JLH. Throughout the trial, the therapists are continuously supervised by JLH in individual and group sessions. All participating therapists had regular contact with the therapy dog for at least 1 year before the start of the trial. To build up a trustful relationship, the therapists trained and played with the therapy dog at least twice a week for 1 year under the supervision of the therapy dog handler (JLH). Furthermore, therapists were trained in dog-assisted psychotherapy (e.g., learnt to read therapy dogs’ stress signals and how to react to them, methods to integrate therapy dogs into psychotherapy).

### Outcomes

#### Sociodemographic information

Participants’ age, gender, and occupation are assessed during the diagnostic interview.

#### Primary outcomes

##### State anxiety

To measure state anxiety, we employ the German version of the State-Trait-Anxiety Inventory (STAI^43^). The questionnaire to assess state anxiety (STAI-S) consists of 20 items (e.g., “I am worried”) that are rated on a four-point Likert scale. The higher the sum score (with values between 20 and 80), the higher the respondent’s state anxiety.

State anxiety is assessed before the treatment (pre treatment), after completion of all steps with the first spider (post first spider), after completion of all steps with the second spider (post second spider), and after completion of all steps with the third spider or after the maximum treatment session duration of 4 hours was reached (post treatment).

##### Positive and negative affect

To measure positive and negative affect, we employ the German version of the Positive and Negative Affect Schedule (PANAS^44^). The PANAS consists of 20 items (10 for positive affect, e.g., “proud” and 10 for negative affect, e.g., “ashamed”) that the participants are asked to rate on a five-point Likert scale based on how they currently feel. The higher the sum score of the positive affect items (with values between 10 and 50), the higher the participant’s positive affect. The higher the sum score of the negative affect items (with values between 10 and 50), the higher the participant’s negative affect.

Positive and negative affect are assessed at the same time points as state anxiety, that is, before the treatment (pre treatment), after completion of all steps with the first spider (post first spider), after completion of all steps with the second spider (post second spider), and after completion of all steps with the third spider or after the maximum treatment session duration of 4 hours was reached (post treatment).

##### Fear and avoidance of spiders

As a self-report measure of fear of spiders, we employ the German version of the FSQ,^45^ the ‘Fragebogen zur Angst vor Spinnen’.^46^ This questionnaire consists of 18 items (e.g., “If I saw a spider now I would be afraid of it.”) that are rated on a seven-point Likert scale. The higher the sum score (with values between 0 and 108), the higher the respondent’s fear of spiders.

As a behavioural measure of fear and avoidance of spiders, we employ the Behavioral Approach Test (BAT^47^). For the BAT, an ordinary living house spider (Tegenaria atrica; around five cm in size, including legs) is placed in a sealed, transparent plastic container on top of a table at the far end of a room, around 6 m away from the door. The participants are asked to enter the room, approach the container, open it, and to hold the spider in their hand(s) for at least 20 s. The participants are motivated to complete this task as far as possible. However, it is emphasised that they can stop at any point. The performance in the BAT is scored between 0 and 12 (0=participant refuses to enter the room, 1=participant stops 5 m from the container, 2=participant stops 4 m from the container, 3=participant stops 3 m from the container, 4=participant stops 2 m from the container, 5=participant stops 1 m from the container, 6=participant stops close to the table with the container, 7=participant touches the container, 8=participant removes the lid, 9=participant puts a hand in the container, 10=participant touches the spider with one finger, 11=participant holds the spider less than 20 s, and 12=participant holds the spider for at least 20 s).

Both the FSQ and the BAT are completed before treatment allocation during the diagnostic interview (baseline), immediately after completion of the treatment (post treatment), and during the 2 weeks in-person follow-up (follow-up 1). The FSQ is additionally completed during the 3-month online follow-up (follow-up 2).

### Secondary outcomes

#### Therapy motivation

To assess the participants’ therapy motivation, we ask them to rate the item “I am motivated to tackle my fear of spiders.” on the seven-point Likert scale of the Fear of Spiders Screening (see the *Additional measures* section below).

The participants rate this item twice: during the diagnostic interview, that is, before the treatment allocation (baseline) and before the treatment (pre treatment).

#### Anticipatory anxiety

To assess the participants’ anticipatory anxiety with regards to the exposure therapy, we ask them to rate the item “When I imagine the upcoming therapy for my fear of spiders, I already feel tense, nervous, and anxious.” on the seven-point Likert scale of the Fear of Spiders Screening (see the *Additional measures* section below).

The participants rate this item twice: during the diagnostic interview, that is, before treatment allocation (baseline) and before the treatment (pre treatment).

#### Fear and avoidance of spiders during treatment as a measure of learning

To briefly assess the fear and avoidance of spiders during the course of the treatment as a measure of learning, we employ the Brief Fear and Avoidance of Spiders Measure (BFASM), which we derived from the Fear of Spiders Screening (see the *Additional measures* section below). In the BFASM, participants are shown a picture of the spider employed in the BAT (an ordinary living house spider, Tegenaria atrica, around 5 cm in size, including legs) sitting on the palm of one of the therapists (only the hand and the lower part of the therapist’s arm are visible) and are asked to rate the two items “If I encountered this spider right now, I would be scared” and “If I encountered this spider right now, I would avoid it” on the seven-point Likert scale of the Fear of Spiders Screening. Higher scores indicate higher fear and higher avoidance, respectively.

The BFASM is completed at the same time points as the STAI-S and the PANAS, that is, before the treatment (pre treatment), after completion of all steps with the first spider (post first spider), after completion of all steps with the second spider (post second spider), and after completion of all steps with the third spider or after the maximum treatment session duration of 4 hours was reached (post treatment).

#### Treatment satisfaction and therapy alliance

To measure treatment satisfaction and therapy alliance (as well as problem activation and mastery), we employ the patient version of the Mainz Hourly Assessment Form (‘Mainzer Stundenbeurteilungsbogen’, MSB^48^). The MSB consists of 15 items (five for therapy alliance, e.g., “My therapist is interested in how I am doing.”, five for problem activation, e.g., “I was emotionally involved.”, and five for mastery, e.g., “I have received help in overcoming my difficulties.”) that are rated on a seven-point Likert scale. All 15 items load on the common factor treatment satisfaction. The higher the sum score of the respective items, the higher the respondent’s treatment satisfaction, therapy alliance, problem activation or mastery. For the purpose of our study, we slightly adapted the instructions of the MSB. In our trial, instead of being asked to refer to the last five sessions when completing the MSB (as in the original version), respondents will either be asked to refer to the session that they just finished (after the preclinical interview and psychoeducation and after the treatment) or to refer to both sessions, that is, the preclinical interview and psychoeducation and the treatment (in the two follow-ups).

Participants complete the MSB four times: after the preclinical interview and psychoeducation (post education), immediately after the treatment (post treatment), during the 2-week in-person follow-up (follow-up 1), and during the 3-month online follow-up (follow-up 2).

### Additional measures

#### Fear of Spiders Screening (SAS)

As a screening instrument for fear of spiders, we employ the Fear of Spiders Screening (‘Spinnenangst Screening’, SAS^46^) during the telephone screening. This screening questionnaire consists of four items (e.g., “I avoid spiders.”) that are derived from the relevant DSM-IV (Diagnostic and Statistical Manual of Mental Disorders 4th edition) criteria for a spider phobia diagnosis and are rated on a seven-point Likert scale. The higher the sum score (with values between 0 and 24), the higher the respondent’s fear of spiders.

#### Short version of the Diagnostic Interview for Mental Disorders (Mini-DIPS)

To assess current and lifetime mental health disorders, we employ the short version of the Diagnostic Interview for Mental Disorders (‘Diagnostisches Kurzinterview bei psychischen Störungen’; Mini-DIPS Open Access^49 50^) during the diagnostic interview. The Mini-DIPS is a structured clinical interview that allows for an efficient yet reliable assessment of mental disorders according to DSM-5 and ICD-10 (International Classification of Diseases 10th edition).

#### Subjective Units of Disturbance (SUDS)

To measure current anxiety, we verbally employ the Subjective Units of Disturbance (SUDS;^51^ also called ‘subjective anxiety scale’^51^ ^p 116^) during the treatment. In the SUDS, participants are given the following instructions: “Think of the worst anxiety you have ever experienced, or can imagine experiencing, and assign to this the number 100. Now think of the state of being absolutely calm and call this zero. Now you have a scale. On this scale how do you rate yourself at this moment?”^51^ ^p 116^ Note that we do not plan to analyse the SUDS ratings but use them as a criterion to advance to the next step during the treatment (see the description of the interventions in the *Participant timeline* section below).

#### Pet Attitude Scale (PAS)

To measure attitude towards pets, we employ the German translation of the Pet Attitude Scale (PAS^52^), the German Pet Attitude Scale.^53^ The PAS consists of 18 items (e.g., “I love pets.”) that are rated on a seven-point Likert scale. The higher the sum score (with values between 18 and 126), the more positive the respondent’s attitude towards pets. The participants complete the PAS once during the diagnostic interview.

#### State-Trait-Anxiety Inventory-Trait (STAI-T)

To measure trait anxiety, we employ the German version of the STAI.^43^ The questionnaire to assess trait anxiety (STAI-T) consists of 20 items (e.g., “I worry too much over something that really doesn't matter”) that are rated on a four-point Likert scale. The higher the sum score (with values between 20 and 80), the higher the respondent’s trait anxiety. The participants complete the STAI-T once during the diagnostic interview.

#### Threat expectancy, threat occurrence, and adjusted threat expectancy

Throughout the treatment, participants’ threat expectancy and adjusted threat expectancy as well as the perceived threat occurrence will be assessed by means of single-item questions, the details, analyses, and results of which will be reported in an article separate from the article in which the results regarding our hypotheses stated above will be reported. The preregistration of our hypotheses and planned analyses regarding these single-item questions can be found here: https://aspredicted.org/2QK_K1N.

#### Maximum anxiety during the treatment and anxiety immediately before the end of the treatment

Throughout the treatment, participants’ maximum anxiety level during the work with each spider and their anxiety level immediately before the end of the work with each spider will be assessed by means of single-item questions, the details, analyses, and results of which will be reported in an article separate from the article in which the results regarding our hypotheses stated above will be reported. The preregistration of our hypotheses and planned analyses regarding these single-item questions can be found here: https://aspredicted.org/2QK_K1N.

#### Saliva samples

To measure salivary cortisol as a biomarker of stress, we collect saliva samples from the therapists and participants by means of Salivettes (Sarstedt AG) at the same time points as the STAI-S, the PANAS, and the BFASM are conducted, that is, before the treatment (pre treatment), after completion of all steps with the first spider (post first spider), after completion of all steps with the second spider (post second spider), and after completion of all steps with the third spider or after the maximum session duration of 4 hours was reached (post treatment). Further details, analyses and results will be reported in an article separate from the article in which the results regarding our hypotheses stated above will be reported.

### Data collection methods

Data collection (except for the saliva samples) is done by means of the software Qualtrics (Qualtrics, Provo, Utah) on tablet computers. To promote participant retention, the in-person follow-up (see the *Participant timeline* section below) is conducted by the respective participant’s therapist and the phone call for the online follow-up (see the *Participant timeline* section below) is made and the email containing the link for the online follow-up is sent by the respective participant’s therapist.

### Harms

We do not expect that the participants suffer any harms due to their participation in our trial.

### Participant timeline

Following an initial telephone screening, trial participation consists of five sessions: (1) diagnostic interview and therapist introduction, (2) preclinical interview and psychoeducation, (3) the one-session treatment, (4) an in-person follow-up 2 weeks after the treatment, and (5) an online follow-up 3 months after the treatment. The sessions (1) to (4) are conducted at the psychotherapy outpatient unit of our university. Note that, in order to have sufficient time to conduct all measures in addition to the 3-hour treatment itself, we set the maximum duration of the treatment session to 4 hours (i.e., 3 hours for the treatment and a total of 1 hour to conduct all measures).

**Figure 1:**
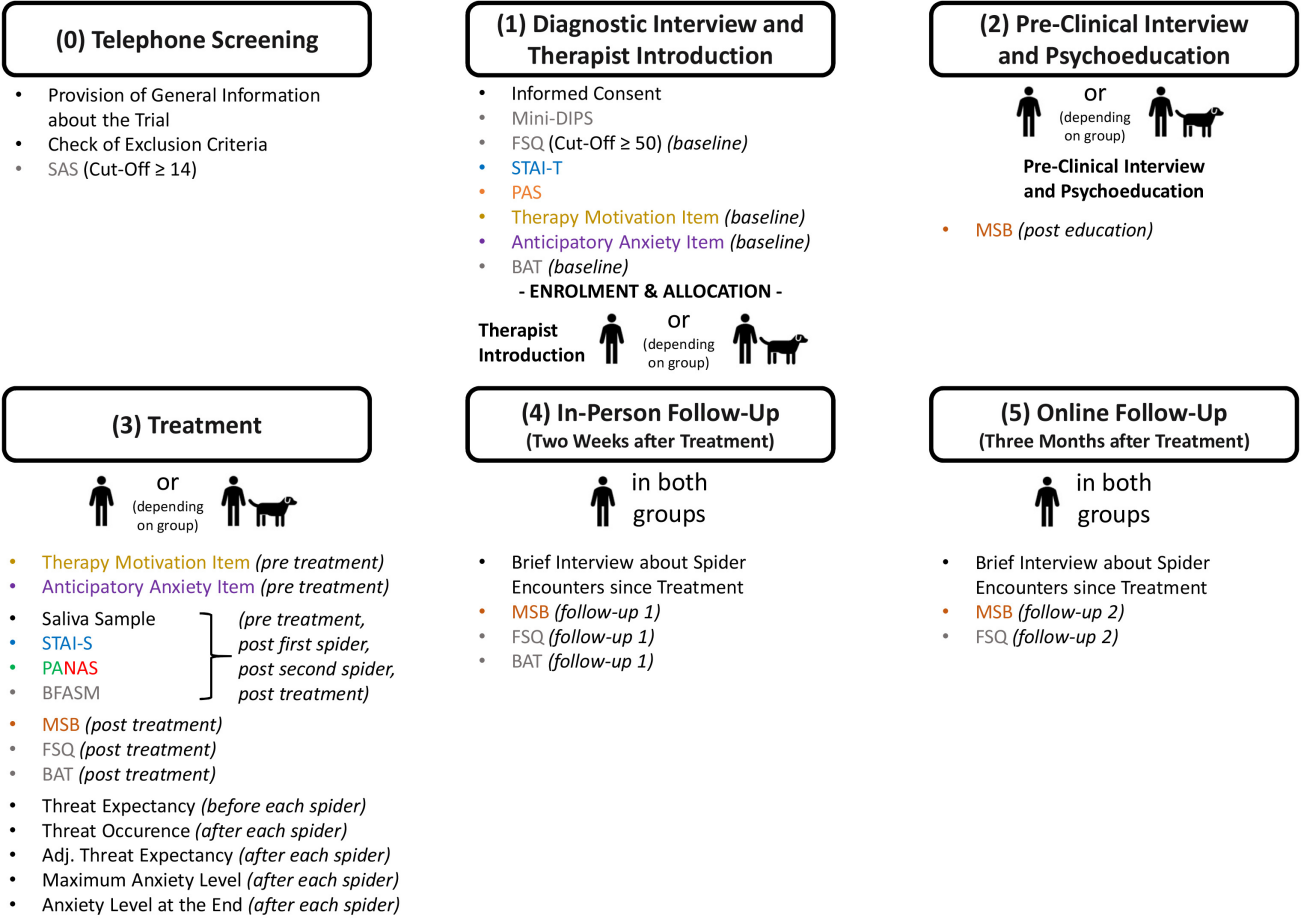
*Trial Procedure (Schedule of Enrolment, Interventions, and Assessments). *SAS = Fear of Spiders Screening. Mini-DIPS = Short version of the Diagnostic Interview for Mental Disorders. FSQ = Fear of Spiders Questionnaire. STAI-T = State-Trait-Anxiety Inventory-Trait. PAS = Pet Attitude Scale. BAT = Behavioral Approach Test. MSB = Mainz Hourly Assessment Form. STAI-S = State-Trait-Anxiety Inventory-State. PANAS = Positive and Negative Affect Schedule. BFASM = Brief Fear and Avoidance of Spiders Measure. For details see the main text.

The procedure of the trial, including information about when the described measures are assessed, is illustrated in figure 1.

### Telephone screening

People who volunteer to participate in our trial are contacted by phone for a brief screening interview. During this interview, general information about the trial is given before the exclusion criteria (i.e., current diagnosis of a mental disorder other than a specific phobia, insect bite allergy, dog hair allergy, fear of dogs, current psychopharmacological treatment, and current psychotherapeutic treatment) are checked. Next, participants are asked to complete the SAS, which is presented orally by the interviewer. Subsequently, an appointment for the diagnostic interview is scheduled with those who score 14 or higher in the SAS (the cut-off employed in previous studies^54^,^56^) and do not fulfil any of the exclusion criteria. All telephone screenings are conducted by a trained person who is neither involved in the therapies nor the follow-ups and is blind regarding the random allocation to the two groups to assure allocation concealment. The telephone screening takes around 10 min to complete.

### Diagnostic interview and therapist introduction

The diagnostic interview is conducted in person at the psychotherapy outpatient unit by the same person who also conducts the telephone screening and takes around 60 min to complete. First, the Mini-DIPS is conducted. Second, participants complete the FSQ, the STAI-T, the PAS, the therapy motivation item, and the anticipatory anxiety item. At this point, participants who score lower than 50 in the FSQ (the cut-off employed in previous studies^54^,^56^) are excluded from the trial. The other participants complete the BAT before subsequently meeting their therapist. The therapist introduces themself, provides information about the upcoming sessions, and schedules appointments for the preclinical interview and psychoeducation, the treatment, and the in-person follow-up.

#### Specification of the two conditions

In the dog group, the therapist is accompanied by the therapy dog when meeting the patient. The therapist introduces themself and the dog and encourages the participant to greet the dog (e.g., pet it, give it a high five). Afterwards, the therapist provides the patient with some information about the therapy dog (e.g., age, sex, origin, character, and preferences of the dog) and the patient is encouraged to play with the dog and to pet it. This approach corresponds to a typical familiarisation with the therapy animal during an animal-assisted intervention and has been applied in previous studies to build up a trustful relationship with a therapy dog.^32^ Subsequently, the therapist provides information about the upcoming sessions and schedules appointments for the preclinical interview and psychoeducation, the treatment, and the in-person follow-up. In the control group, neither is the dog present nor referred to at any point.

#### Preclinical interview and psychoeducation

The preclinical interview and psychoeducation takes place in person at the psychotherapy outpatient unit after the diagnostic session and is led by the therapist. The purpose of this session is twofold: (1) to conduct an individual functional analysis of the participant’s phobia (including a brief biographical analysis and an exploration of the participant’s catastrophic beliefs and safety behaviours; i.e., the preclinical interview part) and (2) to describe the content of and the rationale for the treatment (including general information about spider phobia; i.e., the psychoeducational part). This session lasts about 60 min and is based on the aspects outlined in Öst’s “Manual for the 1-session treatment of specific phobias”.^36^ At the end of the session, the participant and the therapist sign a treatment contract that the participant takes home and the participant completes the MSB.

#### Specification of the two conditions

In the dog group, the therapist is accompanied by the therapy dog and integrates the dog wherever possible. For instance, the dog is repeatedly referred to as an example during discussion and normalisation of safety behaviours and irrational fears, when explaining the usefulness of fear, and in the discussion of pathological fear. To give an example, the therapy dog is afraid of some floor coverings (e.g., slippery surfaces and ventilation grilles). He either avoids these coverings or, if avoidance is not possible, freezes. During the normalisation of safety behaviours and irrational fears, the therapist discusses this example (and others) with the participant. In the control group, by contrast, the therapist solely relies on non-dog examples, for instance, the one provided by Öst in his manual.^36^ Moreover, in the dog group, the participant is free to interact with the dog (e.g., pet the dog, play with the dog) at the beginning and at the end of the session. Moreover, at the end of the session, the treatment contract is signed by the participant, the therapist, and the dog (the dog signs by means of a stamp of its paw). To further strengthen the relationship between the participant and the dog (as perceived by the participant), a polaroid photo of the two is added to the contract that the participant takes home. In the control group, neither is the dog present nor referred to at any point and the contract is signed solely by the therapist and the participant.

### Treatment

The one-session treatment^12^,^14^ takes place in-person at the psychotherapy outpatient unit and follows the procedure outlined in Öst’s “Manual for the 1-session treatment of specific phobias”.^36^ Before the start of the treatment, the participant completes the therapy motivation item and the anticipatory anxiety item. Next, the therapist and the participant provide saliva samples and the participant completes the STAI-S, the PANAS, and the BFASM. Subsequently, the treatment starts. During the treatment, the participant is gradually exposed to three spiders of increasing size (the spiders are indigenous to Germany and range from approximately 1 cm to approximately 5 cm in size, including legs) by means of several exposure steps of increasing difficulty. In detail, the first step comprises catching the smallest spider with a glass and a postcard. The second step comprises touching the smallest spider with the index finger. The third step comprises the smallest spider walking on the participant’s hand. The fourth step comprises the smallest spider walking on the participant’s body. For each of the steps, the therapist first explains and demonstrates it to the participant, before subsequently asking the participant to carry out the step as well. Each step is repeated until the participant’s current anxiety is reduced by 50% in the SUDS. After completion of the fourth step, saliva samples are collected from the therapist and the participant and the participant completes the STAI-S, the PANAS, and the BFASM. Subsequently, this procedure (i.e., the four steps, the saliva collection, and the completion of the STAI-S, the PANAS, and the BFASM) is repeated with the medium-sized and, afterwards, with the largest spider. The session ends after the completion of all steps with all three spiders or after a maximum of 4 hours (i.e., 3 hours for the treatment and a total of 1 hour to conduct all measures). Right before starting to work with each spider, the participant reports their threat expectancy and right after completion of all four steps with the respective spider, the participant reports their adjusted threat expectancy, the perceived threat occurrence, their maximum anxiety level during the work with the spider, and their anxiety level immediately before the end of the work with the spider. Throughout the whole treatment, the therapist challenges the participant’s dysfunctional beliefs and provides corrective information about spiders and their behaviour. Immediately after the treatment, the participant completes the MSB, the FSQ, and the BAT. Eventually, the therapist explicitly encourages the participant to use upcoming spider encounters for training purposes to consolidate what has been learnt.

#### Specification of the two conditions

In the dog group, the therapist is accompanied by the therapy dog and integrates the dog wherever possible. For instance, as during the preclinical interview and psychoeducation, the dog is referred to as an example during the normalisation of safety behaviours and irrational fears and when the usefulness of fear is re-emphasised. Further, the dog is actively integrated to reinforce positive developments in the participant’s interaction with the spiders. For example, when ‘celebrating’ significant steps during the exposure, the dog is involved. In case the participant fixates on the spider as a safety behaviour, they are encouraged to look at the dog instead (which works much better than looking at the therapist or at a certain point in the room). Moreover, the participant is encouraged to interact and to relax with the dog during the short breaks in-between two consecutive spiders. In addition, the participant is free to interact with the dog throughout the session. Importantly, the therapist takes utmost care that the participant does not use these interactions with the dogs to cognitively or behaviourally avoid the spiders and discusses this with the participant if necessary. The dog is present during the work with the first and the third spider but has a break in a different room during the work with the second spider. The dog is free to move throughout the treatment, but most of the time lies in its basket next to the patient. In the control group, neither is the dog present nor referred to at any point.

### In-person follow-up (2 weeks after the treatment)

The first follow-up takes place in person at the psychotherapy outpatient unit, 2 weeks after the treatment. Here, the dog is not present in either group, and the therapist first briefly interviews the participant about how their spider encounters went since the treatment and gives the participant the opportunity to discuss these encounters. Subsequently, the participant completes the MSB, followed by the FSQ, and, eventually, the BAT. To promote participant retention, the in-person follow-up is conducted by the respective participant’s therapist.

#### Specification of the two conditions

The control group and the dog group do not differ with regards to the in-person follow-up.

### Online follow-up (3 months after the treatment)

For the online follow-up, the participant is contacted by phone 3 months after the treatment. The therapist first briefly interviews the participant about how their spider encounters went since the treatment and gives the participant the opportunity to discuss these encounters. The participant then completes the MSB, followed by the FSQ, the link to which is sent to them via email. To promote participant retention, the phone call for the online follow-up is made and the email containing the link is sent by the respective participant’s therapist.

#### Specification of the two conditions

The control group and the dog group do not differ with regard to the online follow-up.

### Sample size

A priori, we calculated the sample size using G*Power 3.1.9.7^57^ to detect a main effect for a group of the size *f*=0.25 in a 2×4 mixed analysis of variance (ANOVA) with the between-subjects factor group (dog group vs. control group), the within-subjects factor time (pretreatment, postfirst spider, postsecond spider, posttreatment), and state anxiety or positive affect as the dependent variables, with a power of 0.80 and a correlation of 0.5 between the observations at an alpha level of 0.05. The effect size estimates are based on the results from our previous study.^32^ This sample size calculation yielded a total sample size of *N*=82 participants (i.e., *n*=41 per group), which we decided to increase to *N*=88 (i.e., *n*=44 per group). Note that with this sample size, our trial has a power greater than 0.99 to detect a medium-sized interaction effect for group X time in the ANOVA, an effect we found previously for state anxiety as the dependent variable.^32^ Importantly, a potential reduction in state anxiety and increase in positive affect in the dog group can only be seen as beneficial if the treatment in the dog group is not inferior to the treatment in the control group (see H5). Thus, it is important to assess the non-inferiority of the treatment in the dog group. Using the approach described by Walker,^58^ given the target sample size of *N*=88, assessments of non-inferiority by means of the FSQ score at an alpha level of 0.05 have a power of around 0.96. Assessment of non-inferiority by means of the BAT score at an alpha level of 0.05 has a power greater than 0.99. Note that these computations require estimates for the standard deviation (*SD*) in the FSQ and the BAT as well as inferiority margins. As estimates for the *SD*, we used the weighted mean of spider phobics’ *SD* observed in previous studies (i.e., 15.5 in the FSQ^46 54 55^ and 2.1 in the BAT^59 60^). For the inferiority margin—the maximum reduction in effectiveness one is willing to accept while still considering the treatments to be equal^58^—we deem the critical difference—the maximum difference between two scores (in the same test, completed by the same person) that are unlikely (with ‘unlikely’ specified by the alpha level) to be solely due to measurement error^61^—to be the best proxy. For the FSQ, we could compute this critical difference. At an alpha level of 0.05, this critical difference is slightly above 12.30 (see the *Statistical methods* section below for the exact computation). For the BAT, to the best of our knowledge, no reliability estimates are available in the literature. Thus, we could not compute the critical difference. As an alternative, we set the inferiority margin to 2, the field’s standard cut-off for a clinically relevant change in the BAT (e.g., Grill *et al.*, 2024, Andersson *et al*., 2009, and Öst *et al., 1998*^59 62 63^).

### Recruitment

We advertise the trial in Germany via newspaper and radio advertisements as well as via flyers distributed at the campus of our university, in several health institutions, and on social media as well as via an announcement by the press office of our university.

### Randomisation: sequence generation, allocation concealment mechanism and implementation

All participants who give written informed consent for participation and who are eligible for inclusion are randomly assigned to either the one-session in vivo exposure treatment of spider phobia with a therapy dog (dog group) or the one-session in vivo exposure treatment for spider phobia without a dog (control group) with a 1:1 allocation as per a computer-generated randomisation schedule. The assignment of participants is done by ME, who is not involved in the screening, the diagnostics, the treatment or any other step of the trial. Allocation concealment is ensured, as the group assignment is not revealed until the participant has been recruited into the trial, that is, after the diagnostic interview (encompassing all baseline measurements) has been completed. The group assignment for a given participant is requested by the person responsible for the respective telephone screening and diagnostic interview (who is not involved in any other part of the trial and who is blind to the allocation). In return, the respective therapist receives a closed envelope containing the group assignment and subsequently meets the participant for the therapist introduction with the dog (dog group) or without the dog (control group).

### Blinding

Due to the nature of the intervention, neither participants nor therapists can be blinded once participants are allocated to one of the two groups. However, the person conducting the telephone screening and diagnostic interview is blind to the allocation, participants are blind to the hypotheses and the respective other groups, and once data collection is completed, an employee outside the research team will feed data into the computer so that the researchers can analyse data without having access to information about the allocation.

### Data management

Data collection (except for the saliva samples) is done by means of the software Qualtrics (Qualtrics, Provo, Utah) on tablet computers. Following common open science practices, data and analysis code will be made publicly available in anonymised form on the Open Science Framework (osf.io). The results of the trial will be published as a research article (including any unforeseen events and deviations from registrations; the results of the saliva sample and the results of the threat expectancy, threat occurrence, adjusted threat expectancy, maximum anxiety during the treatment, and anxiety immediately before the end of the treatment items will be reported in separate research articles).

### Statistical methods

#### Analyses of demographic information

To assess potential differences in age between the groups, we will compute a Bayesian independent samples *t*-test with age as the dependent variable. Note that this allows us to gauge evidence for the null hypothesis that both groups are equal regarding their age.

### Analyses of primary outcomes

#### State anxiety

To analyse the effects of the treatments on state anxiety, we will compute a 2×4 mixed ANOVA with the between-subjects factor group (dog group vs. control group), the within-subjects factor time (pre treatment vs. post first spider vs. post second spider vs. post treatment), and the sum score of the STAI-S as the dependent variable.

#### Positive and negative affect

To assess the effects of the treatments on positive affect and negative affect, we will conduct two 2×4 mixed ANOVAs with the between-subjects factor group (dog group vs. control group), the within-subjects factor time (pre treatment vs. post first spider vs. post second spider vs. post treatment), and the sum score of the positive affect items of the PANAS and the sum score of the negative affect items of the PANAS as the dependent variable, respectively.

#### Fear and avoidance of spiders

To assess potential baseline differences in fear and avoidance of spiders between the dog group and the control group, we will compute two Bayesian independent samples *t*-tests. One with the FSQ score at baseline and one with the BAT score at baseline as the dependent variable. Note that this allows us to gauge evidence for the null hypothesis that both groups are equal regarding their fear and avoidance of spiders at baseline.

To assess the non-inferiority of the treatment in the dog group, we will follow the approach outlined by Walker.^58^ Precisely, for the FSQ, we will compute the following 95% CI at post treatment, follow-up 1, and follow-up 2:


$$\left(\mu_{dog\;group}-\;\mu_{control\;group}\right)\pm 1.96\sqrt{\frac{\sigma_{dog\;group}^{2}}{n_{dog\;group}}+\;\frac{\sigma_{control\;group}^{2}}{n_{control\;group}}}$$

where $\mu_{doggroup}$ and $\mu_{controlgroup}$ are the respective mean FSQ scores in the dog group and the control group, respectively; $\sigma_{doggroup}^{2}$ and $\sigma_{controlgroup}^{2}$ are the respective variances of the FSQ scores in the dog group and the control group, respectively, and $n_{doggroup}$ and $n_{controlgroup}$ are the respective numbers of participants in the dog group and the control group, respectively. Separately for each of the three time points, we will then assess whether the complete 95% CI is within the non-inferiority zone. For the FSQ, as higher scores reflect higher fear and anxiety, the non-inferiority zone is defined as (-∞, *d*_NI_], where *d*_NI_ is the inferiority margin, that is, the maximum reduction in effectiveness one is willing to accept while still considering the treatments to be equal.^58^ We deem the critical difference Δ_crit_ of the FSQ—the maximum difference between two FSQ scores (by the same person) that is unlikely (with ‘unlikely’ specified by the alpha-level) to be solely due to measurement error^61^—to be the best proxy for the inferiority margin *d*_NI_. At an alpha level of 0.05, the critical difference is computed as follows:^56^


$$\Delta_{crit}=1.96*\;s_{x}*\;\sqrt{2*\left(1-r_{tt}\right)}$$

where *s*_x_ is the SD of the test scores and *r*_tt_ is the reliability of the test. With the values reported by Rinck and colleagues,^46^ this formula yields a critical difference of Δ_crit_≈12.30 (rounded down to be more conservative). Thus, we set the inferiority margin to *d*_NI_=12.30, which results in a non-inferiority zone of (-∞, 12.30].

For the BAT, we will compute the 95% CI at post-treatment and follow-up 1, using the same formula as for the FSQ and using the BAT scores and *SD* instead of the FSQ scores and *SD*. To the best of our knowledge, there are no reliability estimates for the BAT reported in the literature. Thus, we could not compute the critical difference in the BAT. As an alternative, we set the inferiority margin *d*_NI_ to 2, the field’s standard cut-off for a clinically relevant change in the BAT (e.g., Grill *et al.*, 2024, Andersson *et al*., 2009, and Öst *et al., 1998*^59 62 63^). As higher BAT scores reflect less fear and avoidance, this results in a non-inferiority zone of [−2, ∞).

To assess the efficacy of the treatments measured by means of the FSQ, we will compute two repeated measures ANOVAs with the factor time (baseline vs. post treatment vs. follow-up 1 vs. follow-up 2) and the FSQ score as the dependent variable. One for each of the two groups. We will use simple contrasts to compare the score at post treatment, at follow-up 1, and at follow-up 2 with the score at baseline.

To assess the efficacy of the treatments measured by means of the BAT, we will compute two repeated measures ANOVAs with the factor time (baseline vs. post treatment vs. follow-up) and the BAT score as the dependent variable. One for each of the two groups. We will use simple contrasts to compare the score at post treatment and at follow-up 1 with the score at baseline.

### Analyses of secondary outcomes

#### Fear and avoidance of spiders during treatment as a measure of learning

To assess the effects of the treatments on learning during the treatment, we will conduct two 2×4 mixed ANOVAs with the between-subjects factor group (dog group vs. control group) and the within-subjects factor time (pre treatment vs. post first spider vs. post second spider vs. post treatment). One with the first and one with the second item of the BFASM as the dependent variable.

#### Treatment satisfaction and therapy alliance

To assess potential differences in treatment satisfaction, therapy alliance, problem activation, and mastery between the dog group and the control group, we will compute four multivariate analyses of variance (MANOVAs) with the between-subjects factor group (dog group vs. control group) and the MSB overall score and the three MSB subscale scores as the dependent variables. One for each of the four time points the MSB is answered by the participants (i.e., after the preclinical interview and psychoeducation, after the treatment, and during both of the follow-ups).

#### Anticipatory anxiety

To assess potential differences in anticipatory anxiety between the groups at baseline, we will compute a Bayesian independent samples *t*-test with the anticipatory anxiety item score at baseline as the dependent variable. To assess potential differences in anticipatory anxiety before the start of the treatment between the groups, we will compute an analysis of covariance (ANCOVA) with the anticipatory anxiety item score at baseline as the covariate, the between-subjects factor group (dog group vs. control group), and the anticipatory anxiety item score at pre treatment as the dependent variable.

#### Therapy motivation

To assess potential differences in therapy motivation between the groups at baseline, we will compute a Bayesian independent samples *t*-test with the therapy motivation item score at baseline as the dependent variable. To assess potential differences in therapy motivation before the start of the treatment between the groups, we will compute an ANCOVA with the therapy motivation item score at baseline as the covariate, the between-subjects factor group (dog group vs. control group), and the therapy motivation item score at pre treatment as the dependent variable.

### Analyses of additional outcomes

#### Pet attitude

To assess the influence of the attitude towards pets on the treatment efficacy measured by means of the FSQ, we will compute a multiple regression for each of the time points post treatment, follow-up 1, and follow-up 2, with the FSQ score at baseline and the PAS score as predictors and the FSQ score at the respective time point as the dependent variable.

To assess the influence of the attitude towards pets on the treatment efficacy measured by means of the BAT, we will compute a multiple regression for each of the time points post treatment, follow-up 1, and follow-up 2, with the BAT score at baseline and the PAS score as predictors and the BAT score at the respective time point as the dependent variable.

#### Trait anxiety

To assess potential differences in trait anxiety between the groups at baseline, we will compute a Bayesian independent samples *t*-test with the STAI-T score at baseline as the dependent variable.

### Dealing with missing data

The statistical analyses will be conducted as intention-to-treat analyses. We will deal with missing data by means of multiple imputation.^64^ As a more conservative approach in the assessment of non-inferiority,^58^ we will additionally conduct the non-inferiority analyses as per protocol analyses.

### Data monitoring committee

Given our trials target population (non-critical indications), the characteristics of our interventions (no harm expected), the fact that our trial can be completed in a short time frame (participants are treated for a short time only), and the fact that no interim analyses are planned, no data monitoring committee will be involved in the trial.^65^

### Trial monitoring

Given our trial’s target population (non-critical indications), the characteristics of our interventions (no harm expected), and the fact that our trial can be completed in a short time frame (participants are treated for a short time only), no trial monitoring outside the research team is planned.

## Ethics and dissemination

### Research ethics approval

This trial was approved by the Ethics Committee of the Faculty for Human and Business Sciences of Saarland University (reference number 24–11).

### Dissemination policy

The findings from this trial will be disseminated by means of common academic pathways, including peer-reviewed publications and conference presentations.

### Protocol amendments

Any deviations from this study protocol or the preregistrations as well as any adverse events potentially arising in the course of the trial will be made explicit in the publication of the trial results.

### Consent or assent

All participants provided written informed consent prior to the inclusion into the trial.

### Confidentiality

We will store all records that contain names or other personal identifiers, such as informed consent forms, in locked file cabinets in areas with limited access. Locally, we will store the anonymised data on password-protected hard drives in a separate locked cabinet.

### Ancillary and post-trial care

We do not plan ancillary or post-trial care.

## Supplementary material

10.1136/bmjopen-2025-101648 online supplemental file 1
