# Supplementary material for: Support on four paws—does the integration of a therapy dog reduce anxiety and increase positive affect in spider phobics during in vivo exposure therapy?: study protocol for a parallel randomised controlled trial of two groups to compare one-session in vivo exposure treatment of spider phobia with and without a therapy dog
Source: BMJ Open. 2025 Jul 15;15(7):e101648. doi: 10.1136/bmjopen-2025-101648 (PMC12265806; doi:10.1136/bmjopen-2025-101648)
Supplement: online supplemental file 1 [file bmjopen-15-7-s001.docx]

**Datenschutzrechtliche Aufklärung und Einwilligung inkl. Information gem. Art.13 EU-DSGVO**

**1. Ausführliche Beschreibung des Forschungsvorhabens**
Wir freuen uns sehr, dass Sie Interesse haben, an unserer Studie teilzunehmen!

Ziel unserer Studie ist es, den Einfluss einer Konfrontationstherapie auf eine vorliegende Spinnenphobie zu testen. Konfrontationstherapie bedeutet die schrittweise Konfrontation mit dem angstauslösenden Reiz, in diesem Fall einer Spinne.

Die gesamte Studie umfasst ein Telefonscreening, 4 Präsenztermine an der *UNIVERSITY* und eine abschließende Onlinebefragung. Der heutige Termin beinhaltet ein Vorgespräch und das Ausfüllen einiger Fragebögen. Beim zweiten Termin werden Sie einige Informationen zu Spinnenphobie, Konfrontationstherapie und dem genauen Ablauf der Therapie erhalten. Der dritte Termin ist dann das Kernstück, die dreistündige Konfrontationstherapie. Im Anschluss an die Therapie wird noch eine Nachbesprechung und Erfolgsauswertung stattfinden. Diese wird in einem vierten Präsenztermin nach einer Woche wiederholt. Eine kurze Onlinebefragung zum weiteren Erfolgserhalt wird dann ca. 3 Monate später erfolgen.

Die Studie wird von geschulten Psychologiestudent*innen und Psychotherapeut*innen in Ausbildung unter stetiger Supervision einer erfahrenen Psychotherapeutin durchgeführt.

Die Teilnahme an der Studie ist freiwillig. Sie können jederzeit und ohne Angabe von Gründen Ihre Einwilligung zur Teilnahme an dieser Studie widerrufen, ohne dass Ihnen daraus Nachteile entstehen.

**2. Inhalt und Zweck der Studie**
Ziel unserer Studie ist es, den Einfluss einer Konfrontationstherapie auf eine vorliegende Spinnenphobie zu testen. Die von Ihnen erhobenen Daten werden ausschließlich zu wissenschaftlichen Zwecken, konkret der Forschung im Bereich der Konfrontationstherapie verwendet.

**3. Betroffener Personenkreis**
Teilnahmeberechtigt sind alle volljährigen Personen mit Spinnenphobie, die keine aktuelle psychische Diagnose außer einer spezifischen Phobie haben, sich aktuell weder einer pharmakologischen Therapie noch einer Psychotherapie unterziehen, keine Hundehaarallergie, keine Insektenstichallergie und keine Angst vor Hunden haben.

**4. Zu erhebende Daten**
Im Rahmen der Studie werden Daten zu Symptomen von Spinnenphobie, subjektivem Erleben und subjektiven Einstellungen der Proband*innen erhoben.

**5. Analyseergebnisse der Daten**
Ihre Angaben werden streng vertraulich behandelt. Die erhobenen Daten sind anonymisiert. Wir sind nur an der statistischen Auswertung der Ergebnisse von Gruppen von Teilnehmerinnen und Teilnehmern, nicht spezifisch an Analysen einzelner Personen interessiert.

**6. Lagerung und Weitergabe der Daten**
Die Ergebnisse dieser Studie können als wissenschaftliche Publikation veröffentlicht und/oder als „Open Data“ in einem sicheren Repositorium (wie z. B. Open Science Framework, https://osf.io/) zugänglich gemacht werden, d.h. dass auch andere Forscherinnen und Forscher, die nicht unmittelbar an der Datenerhebung beteiligt waren, diese Daten zur Forschung nutzen können. Dieses Vorgehen entspricht den Empfehlungen der Deutschen Forschungsgemeinschaft (DFG) zur Qualitätssicherung in Bezug auf Nachprüfbarkeit und Reproduzierbarkeit wissenschaftlicher Ergebnisse.

**7. Beteiligte, Datenflüsse und speichernde Stellen**
Folgende Personen sind primär an der Auswertung der Daten beteiligt:
*ANONYMIZED*

**8. Konkrete Dauer der Speicherung**
Aufgrund der Möglichkeit der Veröffentlichung der erhobenen Daten als wissenschaftliche Publikation und/oder als „Open Data“ in einem sicheren Repositorium (siehe Punkt 6) ist prinzipiell von einer unbegrenzten Speicherung der Daten auszugehen.

**9. Rechtsgrundlage**
Die Rechtsgrundlage zur Verarbeitung der genannten personenbezogenen Daten bildet die Einwilligung gemäß Art. 6 (1) Buchstabe a EU-DSGVO im zweiten Teil dieses Dokumentes.

**10. Widerruf seitens des/der Betroffenen**
Sie haben das Recht, jederzeit die datenschutzrechtliche Einwilligung zu widerrufen. Durch den Widerruf der Einwilligung wird die Rechtmäßigkeit der aufgrund der Einwilligung bis zum Widerruf erfolgten Verarbeitung nicht berührt (Widerruf mit Wirkung für die Zukunft). Daten, die im Rahmen von Forschung bereits in Statistiken etc. eingeflossen sind oder deren Löschung anderweitig mit einem unverhältnismäßigen Aufwand verbunden wäre, können in der Regel nicht rückwirkend herausgenommen werden. Richten Sie den Widerruf an den Verantwortlichen. Ihnen entstehen durch den Widerruf keine Nachteile.

**11. Namen und Kontaktdaten des Verantwortlichen**
Die Verantwortung für die Verarbeitung der personenbezogenen Daten hat:
*ANONYMIZED*

**12. Kontaktdaten des Datenschutzbeauftragten***ANONYMIZED*

**13. Hinweis auf Rechte des/der Betroffenen**
Gemäß Art. 13 Abs.2 lit. b der Datenschutzgrundverordnung haben Sie das Recht auf
Auskunft (Art 15 DSGVO und §34 BDSG)
Widerspruch (Art. 21 DSGVO 2018 und §36 BDSG)
Datenübertragbarkeit (Art 20 DSGVO)
Löschung (Art 17 DSGVO und §35 BDSG)
Einschränkung der Verarbeitung (Art 18 DSGVO)
Berichtigung (Art 16 DSGVO)

Möchten Sie eines dieser Rechte in Anspruch nehmen, wenden Sie sich bitte an den folgenden Verantwortlichen.
*ANONYMIZED*

Weiterhin haben Sie das Recht, Beschwerde bei der Aufsichtsbehörde einzulegen:
*ANONYMIZED*

**14. Weitergabe von Informationen über die Studie**
Da unsere Erfahrung zeigt, dass ein Weitererzählen der Inhalte einer Studie selbst in kleinem Rahmen die Ergebnisse einer Untersuchung verfälschen kann, bitten wir Sie, über die Inhalte dieser Studie bis Ende des Jahres 2025 absolutes Stillschweigen zu bewahren, um eine Gefährdung der Ziele der Datenerhebung auszuschließen.

Bei Rückfragen können Sie sich an die Projektleitung wenden:
*ANONYMIZED*

**Einwilligungserklärung**

Hiermit bestätige ich, dass ich die Informationen zur Studie aufmerksam gelesen und deren Inhalt (Zielsetzung, Ablauf, Anonymisierung) verstanden habe. Ich hatte die Gelegenheit, alle mich interessierenden zusätzlichen Fragen zu stellen. Es stand mir ausreichend Bedenkzeit zur Verfügung und mir ist bewusst, dass neu auftauchende Fragen jederzeit besprochen werden können.

Mir ist bekannt, dass ich jederzeit ohne Angabe von Gründen und ohne Inkaufnahme von Nachteilen von den Untersuchungen zurücktreten kann.

Ich kann mich zu jedem Zeitpunkt mit weiteren Fragen an den Versuchsleiter wenden.

Ich erkläre mich hiermit freiwillig bereit und damit einverstanden, an der Studie teilzunehmen.

*LOCATION*, __________ _______________________________ _______________________

(Datum) (Name, Vorname Proband*in) (Unterschrift Proband*in)

_____________________________ ________________________________

(Name, Vorname Versuchsleitung) (Unterschrift Versuchsleitung)
