# Supplementary material for: Support on four paws—does the integration of a therapy dog reduce anxiety and increase positive affect in spider phobics during in vivo exposure therapy?: study protocol for a parallel randomised controlled trial of two groups to compare one-session in vivo exposure treatment of spider phobia with and without a therapy dog
Source: BMJ Open. 2025 Jul 15;15(7):e101648. doi: 10.1136/bmjopen-2025-101648 (PMC12265806; doi:10.1136/bmjopen-2025-101648)
Supplement: Supplementary file 2 [file bmjopen-2025-101648.reviewer_comments.pdf]

PEER REVIEW HISTORY

BMJ Open publishes all reviews undertaken for accepted manuscripts. Reviewers are asked to complete a checklist review form and are provided with free text boxes to elaborate on their assessment. These free text comments are reproduced below.

ARTICLE DETAILS

Title (Provisional)

Support on four paws – Does the integration of a therapy dog reduce anxiety and increase positive affect in spider phobics during in vivo exposure therapy?: Study protocol for a parallel randomized controlled trial of two groups to compare one-session in vivo exposure treatment of spider phobia with and without a therapy dog

Authors

Braun, Moritz Nicolai; Michael, Tanja; Equit, Monika; Lass-Hennemann, Johanna

---

VERSION 1 - REVIEW

**Reviewer**	**1**
**Name**	**Kroczek, Agnes**
**Affiliation**	**University Hospital Tubingen Department of Internal Medicine**
**Date**	**17-Apr-2025**
**COI**	**None**

The manuscript is very well-written, the approach of the study for a dog-supported fear-extinction is very interesting. Furthermore, the study design is very sound so I recommend it for publication. There are only following points that need to be adressed:

1. Why there is no analysis for the SUD ratings during the exposure session? Actually I would be very interested in these results, this being related to:

2. Within the introduction, the theoretical background of cue exposure should be stated, adressing the point that usually the approach requires anxiety to fully evolve for extinction being most effective. How does the dog integrate into that model and is differencated from a safety signal? If authors hypothesize a biological background (oxytocin?) this should be cleary adressed. If not, please differentiate how a dog dissociates from a mere saftey signal.

Page 25, line 528: There is an "I" to much

**Reviewer**	**2**
**Name**	**Alhaj, Hamid**

**Affiliation**	**University of Sharjah**
**Date**	**22-May-2025**
**COI**	**None**

This study investigates a relevant and timely topic, enhancing the acceptability and effectiveness of exposure therapy by integrating a therapy dog into the treatment process. The focus on spider phobia is appropriate, as it provides a clear, manageable context for assessing the feasibility and preliminary effects of the intervention.

The use of a manualised one-session exposure therapy protocol is a strength, as it is grounded in evidence-based practice. However, the authors should acknowledge as a limitation that this brief format may not reflect typical clinical settings, where exposure therapy is commonly delivered over multiple sessions. Addressing this issue would enhance the study's ecological validity.

The inclusion of saliva sample collection from both patients and therapists is a valuable methodological feature. While it is understandable that full analysis will be reported in a separate publication, the current manuscript would benefit from a brief description of the purpose of these samples, such as measuring cortisol as a biomarker of stress. This would help readers understand the rationale for including biological data.

The plan to assess the therapeutic alliance is another notable strength. However, the manuscript would be improved by including a concise explanation of the theoretical relevance of this construct in the introduction. This would clarify its role in the study's framework and how it may be influenced by the presence of a therapy dog.

A further point to consider is the cultural and religious variability in attitudes toward therapy dogs. The authors should briefly discuss how these factors might affect the generalizability or acceptance of the intervention across different populations.

Specific Comments:

• Referencing "see below": The repeated use of vague references such as "see below" should be replaced with specific section or table numbers to improve clarity and navigation.

• Lines 214–215: The phrase “that do not fulfill any criteria” is unclear and likely a fragment or editing error. It should either be clarified or removed.

• Inclusion/Exclusion Criteria: Presenting inclusion and exclusion criteria in separate paragraphs would improve readability. Also, terms like “current diagnosis of a mental disorder” should be specified using standardised diagnostic criteria (e.g., DSM-5 or ICD-10). Indicating how these diagnoses are assessed (e.g., structured clinical interview) would further strengthen this section.

• Line 333 – DSM-5

VERSION 1 - AUTHOR RESPONSE

Reviewer: 1
Dr. Agnes Kroczek, University Hospital Tubingen Department of Internal Medicine
Comments to the Author:
The manuscript is very well-written, the approach of the study for a dog-supported fear-extinction is very interesting. Furthermore, the study design is very sound so I recommend it for publication. There are only following points that need to be adressed:

<u>**Response:**</u> Thank you very much for your positive feedback. We very much hope that we have sufficiently addressed all your remarks.

1. Why there is no analysis for the SUD ratings during the exposure session? Actually I would be very interested in these results, this being related to:

<u>**Response:**</u> We do not analyze the SUDS ratings during the exposure session as we only collect them orally and use them to advance to the next step during the treatment. We agree that it would be interesting to analyze the SUDS ratings, which is why we discussed collecting and analyzing the SUDS ratings when designing our trial. Eventually, we decided against collecting the SUDS ratings by means of tablet computers (which would be a necessary first step for the analyses) because we (1) did not want the treatment to be disrupted at deciding moments by the tablet-based data collection and (2) wanted to assure maximum flexibility on part of the therapist about the timing of the SUDS, which would greatly reduce comparability of the SUDS results between participants. However, with the single item questions regarding the participants' maximum anxiety level during the work with each spider and their anxiety level immediately before the end of the work with each spider we do collect data on the participants' anxiety (using the same scale as the SUDS) that we plan to analyze.

2. Within the introduction, the theoretical background of cue exposure should be stated, adressing the point that usually the approach requires anxiety to fully evolve for extinction being most effective. How does the dog integrate into that model and is differencated from a safety signal? If authors hypothesize a biological background (oxytocin?) this should be cleary adressed. If not, please differentiate how a dog dissociates from a mere saftey signal.

<u>**Response:**</u> Thank you very much for raising these important aspects.

The mechanisms of change in exposure therapy, including anxiety evolvement (often referred to as initial fear activation), are the subject of current research and the Exposure Therapy Consortium has recently published a research agenda for future research on the mechanisms of change of exposure therapy (Benito et al., 2024 https://doi.org/10.1177/21677026241240727). Interestingly, recent research suggests change in threat expectancy as a central mechanism of exposure therapy (e.g. Pittig et al., 2022 ; Winkler et al., 2025), while initial fear activation as well as within-session and between-session habituation are increasingly being called into question as relevant mechanisms (for a recent review see Knowles & Tolin, 2022 https://doi.org/10.1007/s11920-022-01391-8). To contribute to this ongoing and highly relevant debate, within the framework of our trial, we also collect data on these mechanisms by means of the items on threat expectancy, threat occurrence, and adjusted threat expectancy and the items on maximum anxiety during the treatment and anxiety immediately before the end of the treatment (see the *Additional measures* section in our manuscript). As we (1) plan to report the results from these items in a separate article solely

concerned with the mechanisms of change in exposure therapy and (2) feel that our study protocol is already quite complex, we would like to refrain from delving deeper into the theoretical background of cue exposure and by this into the mechanisms debate in the introduction of our study protocol and to save this for the mentioned separate article.

There is evidence that safety signals hinder fear extinction and impair exposure therapy (e.g., Lovibond et al., 2000 https://doi.org/10.1016/S0005-7967(99)00121-7; see Hermans et al., 2006 https://doi.org/10.1016/j.biopsych.2005.10.006 for a review), which is why it is common practice to avoid the presence of safety signals during exposure therapy. However, recent evidence suggests that social support differs from safety signals in the respect that, although social support also inhibits fear while it is present (just as safety signals do), it reduces fear in the long-term and by that may enhance fear extinction (Hornstein et al., 2018 https://doi.org/10.1177/21677026177430; see Hornstein & Eisenberger, 2021 https://doi.org/10.20900/jpbs.20210010 for a discussion). We delve deeper into this literature and the question as of whether and how a dog differs from a safety signal and from social support in the article, in which we report the findings from our previous laboratory study (the study we describe in our introduction, based our power-calculations on, and in which we compared the dog group not only to a control group but also to a social support group). Given this article, which we currently prepare for publication, and that (1) our study protocol is already quite complex and (2) the quality of a discussion of the relation of dog, safety signal, and social support will suffer when trying to keep the introduction of our study protocol at a reasonable length (especially so with regard to the question as of how the dog-safety signal-social support relation relates to the mechanisms debate), we would like to refrain from delving deeper into this topic in our study protocol.

Within the framework of our present trial, we are agnostic toward the mechanisms underlying the effects of the integration of a dog into therapy. Thus, we do not discuss these mechanisms anywhere in our study protocol.

Page 25, line 528: There is an "I" to much

Response: Thank you for pointing this out. We removed it.

Reviewer: 2
Dr. Hamid Alhaj, University of Sharjah
Comments to the Author:
This study investigates a relevant and timely topic, enhancing the acceptability and effectiveness of exposure therapy by integrating a therapy dog into the treatment process. The focus on spider phobia is appropriate, as it provides a clear, manageable context for assessing the feasibility and preliminary effects of the intervention.

Response: Thank you very much for your positive evaluation.

The use of a manualised one-session exposure therapy protocol is a strength, as it is grounded in evidence-based practice. However, the authors should acknowledge as a limitation that this brief format may not reflect typical clinical settings, where exposure therapy is commonly delivered over multiple sessions. Addressing this issue would enhance the study's ecological validity.

Response: Thank you very much for raising this limitation. We have added it to our "Strengths and limitations" section. We now write:

- *In its briefness, the one-session treatment employed in the present trial may not necessarily reflect typical clinical settings, where exposure therapy is commonly delivered over multiple sessions*

The inclusion of saliva sample collection from both patients and therapists is a valuable methodological feature. While it is understandable that full analysis will be reported in a separate publication, the current manuscript would benefit from a brief description of the purpose of these samples, such as measuring cortisol as a biomarker of stress. This would help readers understand the rationale for including biological data.

<u>**Response:**</u> Thank you very much for pointing this out. We have added the information that we collect the saliva samples for the purpose of measuring salivary cortisol as a biomarker of stress. We now write:

To measure salivary cortisol as a biomarker of stress, we collect saliva samples from the therapists and participants by means of Salivettes (Sarstedt AG) at the same time points as the STAI-S, the PANAS, and the BFASM are conducted, i.e. before the treatment (pre treatment), after completion of all steps with the first spider (post first spider), after completion of all steps with the second spider (post second spider), and after completion of all steps with the third spider or after the maximum session duration of four hours was reached (post treatment). Further details, analyses, and results will be reported in an article separate from the article in which the results regarding our hypotheses stated above will be reported.

The plan to assess the therapeutic alliance is another notable strength. However, the manuscript would be improved by including a concise explanation of the theoretical relevance of this construct in the introduction. This would clarify its role in the study's framework and how it may be influenced by the presence of a therapy dog.

<u>**Response:**</u> We are glad that you agree with us that assessing the therapeutic alliance is interesting. We have added a concise explanation of the theoretical relevance to our introduction. We now write:

In addition, we explore whether participants in the dog group report higher treatment satisfaction than participants in the control group and whether participants in the dog group report a better therapy alliance than participants in the control group. The latter seems especially interesting in light of the importance of the therapeutic alliance for the success of psychotherapy [38] and ongoing research about whether and how the presence of a dog improves the therapeutic alliance [39-41].

A further point to consider is the cultural and religious variability in attitudes toward therapy dogs. The authors should briefly discuss how these factors might affect the generalizability or acceptance of the intervention across different populations.

<u>**Response:**</u> Thank you very much for raising this important aspect. We have added the following to our introduction:

Importantly, differences in the attitude toward pets in general and dogs in particular, potentially due to differences in cultural or religious backgrounds, might influence the acceptance and effects of the integration of a dog into therapy. To be able to address this, we also collect data on the participants' attitude towards pets.

Specific Comments:
• Referencing "see below": The repeated use of vague references such as "see below" should be replaced with specific section or table numbers to improve clarity and navigation.

__Response:__ Thank you very much for pointing this out. We replaced the vague “see below” references with references to specific references. For instance, we now write:

Eligible for inclusion are adults with spider phobia with a score of at least 14 in the Fear of Spider Screening (see the Outcomes section below) and a score of at least 50 in the Fear of Spider Questionnaire (see the Outcomes section below) that do not fulfill any exclusion criteria.

• Lines 214–215: The phrase “that do not fulfill any criteria” is unclear and likely a fragment or editing error. It should either be clarified or removed.

__Response:__ Thank you very much for pointing this out. The word “exclusion” must have gotten lost during the preparation of our manuscript. We have corrected this by adding the word “exclusion” and now write:

Eligible for inclusion are adults with spider phobia with a score of at least 14 in the Fear of Spider Screening (see the Outcomes section below) and a score of at least 50 in the Fear of Spider Questionnaire (see the Outcomes section below) that do not fulfill any exclusion criteria.

• Inclusion/Exclusion Criteria: Presenting inclusion and exclusion criteria in separate paragraphs would improve readability. Also, terms like “current diagnosis of a mental disorder” should be specified using standardised diagnostic criteria (e.g., DSM-5 or ICD-10). Indicating how these diagnoses are assessed (e.g., structured clinical interview) would further strengthen this section.

__Response:__ Thank you very much for this suggestion to improve readability. Using the SPIRIT template to report our study protocol, we now present the eligibility criteria in a separate paragraph, which we further split in two paragraphs concerned with the inclusion and exclusion criteria. Moreover, we specified the term “current diagnosis of a mental disorder”. We now write:

Exclusion criteria comprise the current diagnosis of a mental disorder other than a specific phobia (either already known to the participant prior to their participation in our study or diagnosed by means of the Short version of the Diagnostic Interview for Mental Disorders (Mini-DIPS; see the Additional measures section below for details) during the course of the diagnostic interview), insect bite allergy, dog hair allergy, fear of dogs, current psychopharmacological treatment, and current psychotherapeutic treatment. All participants give their written informed consent to participate in the trial (see appendix).

• Line 333 – DSM-5

__Response:__ Thank you for mentioning this. We have corrected it.
